# A biomechanical investigation of three fixation methods for unilateral denis type II sacral fractures using finite element analysis

**DOI:** 10.3389/fbioe.2025.1631457

**Published:** 2025-08-25

**Authors:** Peishuai Zhao, Renjie Li, Jiaqiang Chen, Yongsheng Wang, Jianzhong Guan, Min Wu

**Affiliations:** Department of Orthopaedics, The First Affiliated Hospital of Bengbu Medical College, Bengbu, China

**Keywords:** biomechanical, sacral fracture, triangular osteosynthesis, S2-alar-iliac screw, finite element analysis

## Abstract

**Objective:**

Due to its inherent high instability, the selection of fixation strategies for unilateral Denis type II sacral fractures remains a controversial challenge in the field of traumatic orthopedics. This study focuses on unilateral Denis type II sacral fractures. By applying three different fixation methods, it aims to explore their biomechanical properties and provide a theoretical basis for optimizing clinical fixation protocols.

**Methods:**

A ligament-intact three-dimensional finite-element model of a right-sided Denis type II sacral fracture, including ipsilateral superior and inferior pubic rami fractures, was generated. Three fixation models were simulated: (1) S1/S2 transiliac-transsacral screw fixation (S1/S2-TTS); (2) unilateral L4/5 triangular osteosynthesis (UTOS); and (3) bilateral S2-alar-iliac screws combined with an iliosacral screw (BS2AI-ISS). Appropriate material properties, boundary conditions, and loading protocols were assigned. A 500 N axial compressive load superimposed with a 7.5 Nm torque was applied to simulate standing position and multiplanar spinal motion. Biomechanical parameters evaluated included vertical sacral stiffness, maximum von Mises stress within implants, and relative interfragmentary displacement (RID) at the fracture site.

**Results:**

Sacrum vertical stiffness: All constructs significantly increased sacrum vertical stiffness compared with the intact model. Normalised stiffness values were 443.18% (S1/S2-TTS), 228.38% (UTOS) and 397.26% (BS2AI-ISS). Maximum implant von Mises stress: Under every loading mode, S1/S2-TTS exhibited the lowest and most evenly distributed stress (range 30.30–49.23 MPa). Maximum stresses ranked from lowest to highest: S1/S2-TTS < BS2AI-ISS < UTOS. Relative interfragmentary displacement: In standing position, mean RID were 0.0313 ± 0.0148 mm (S1/S2-TTS), 0.0736 ± 0.0314 mm (UTOS) and 0.0539 ± 0.0163 mm (BS2AI-ISS). Only the difference between S1/S2-TTS and UTOS reached statistical significance (p = 0.047). Similar patterns were observed in extension, left flexion and left rotation; no significant differences were found in right flexion or right rotation.

**Conclusion:**

The present study demonstrates that BS2AI-ISS provides biomechanical stability comparable to both S1/S2-TTS and UTOS for unilateral Denis type II sacral fractures. Notably, BS2AI-ISS achieves this stability without compromising lumbar motion and irrespective of sacral morphologic variations. These findings suggest that BS2AI-ISS may serve as an effective alternative for managing unilateral Denis type II sacral fractures.

## 1 Introduction

The pelvis serves as the cornerstone connecting the spine and lower extremities, primarily relying on the sacrum and surrounding strong ligaments to bear the downward axial pressure from the spine and the upward counterforce transmitted from the lower limbs (Kweh et al., 2022; Liu et al., 2016b; Pascal-Moussellard et al., 2016). When subjected to shear force over an extended period, both high-energy and low-energy trauma can lead to vertically unstable sacral fractures (Acklin et al., 2018; Endo et al., 2025). Among these, the transforaminal vertically unstable sacral fracture (Denis type II) is one of the severe injuries to the posterior pelvic ring, mostly caused by high-energy trauma (Barber et al., 2023). It can result in pelvic ring collapse, lower limb length discrepancy, and irreversible nerve traction injuries (Xu et al., 2022). Conservative treatment may lead to adverse complications such as fracture redisplacement, hypostatic pneumonia, and bedsores, significantly increasing the disability and mortality rates (Lehman et al., 2012). Therefore, restoring the sacral height and maintaining vertical stability are the core goals of surgery (Liu et al., 2021).

Multiple studies have shown that the use of two transiliac-transsacral screws (TTS) is an effective method to stabilize the fracture (Fu et al., 2014; Turbucz et al., 2023; Zheng et al., 2021; Ziran et al., 2022). However, the sacral dysmorphism rate is as high as 40%, which may increase the risk of screw placement and neurovascular injury (Jäckle et al., 2022; Jeong et al., 2017; Kaiser et al., 2014). Triangular Osteosynthesis (TOS) technique proposed by Schildhauer et al. (2003) offers biomechanical stability comparable to that of TTS, but it requires fixation of the lumbar spine, which affects mobility and can lead to screw protrusion or skin necrosis (Min et al., 2014; Wenning et al., 2021). In recent years, S2-alar-iliac (S2AI) screws have gained popularity for posterior pelvic-ring stabilization (Wakayama et al., 2022). Du et al. (2024) found that bilateral S2AI screw fixation provides superior stability in low-density pelvic models compared to ilioscral screw (ISS) and TTS fixation, and it offers more cortical purchase and fewer skin complications during lumbopelvic fixation, although it still restricts activity in the lumbosacral region.

We therefore propose a novel construct—bilateral S2AI screws combined with an iliosacral screw (BS2AI-ISS)—that theoretically avoids both sacral morphologic constraints and compromise of lumbar mobility. However, it remains unclear whether this new internal fixation method can meet the biomechanical stability required for sacral fractures.

The present finite-element study was designed to compare the biomechanical performance of S1/S2-transiliac-transsacral screws (S1/S2-TTS), unilateral L4/L5-triangular osteosynthesis (UTOS), and BS2AI-S1 constructs in stabilising unilateral Denis type II sacral fractures. Particular attention was paid to fracture-site displacement and implant stress distribution, with the aim of providing a new perspective for the clinical selection of internal fixators.

## 2 Methods

### 2.1 Construction of the intact spino-pelvis finite element model

**FIGURE 1:**
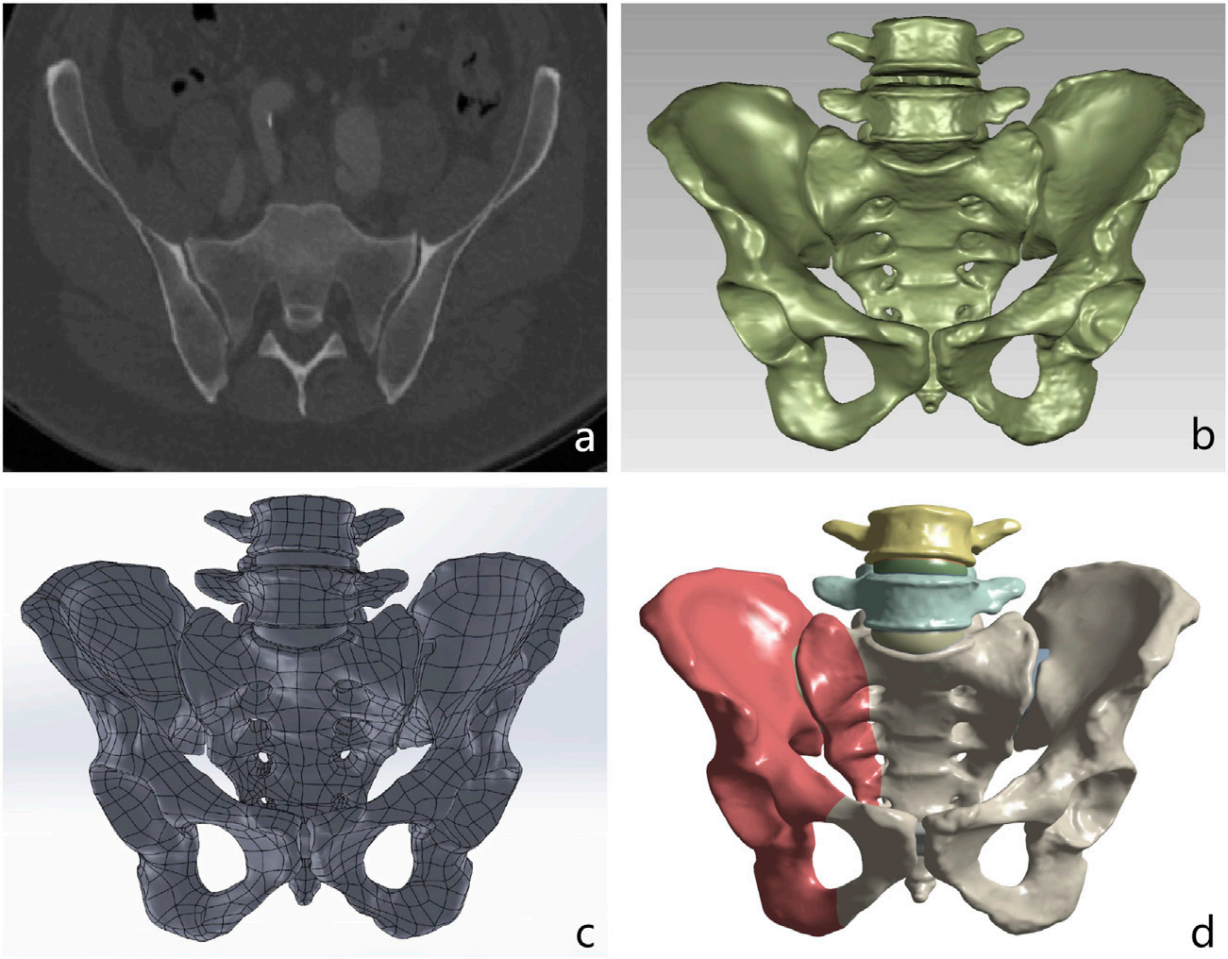
Process of creating a finite element model from CT scan data for biomechanical analysis. **(a)** Original DICOM CT scan image of the pelvis and lumbar spine. **(b)** High-quality surface model after denoising and smoothing in Geomagic Studio. **(c)** Spino-pelvic model with intervertebral discs and cartilage constructed in Solidworks. **(d)** The pelvic Denis type II fracture model imported into the ANSYS software is used to simulate the mechanical analysis under the fracture condition.

The three-dimensional finite element model of the spine-pelvis (L4-L5-Pelvic) was derived from the CT images (64-slice CT scan with a slice thickness of 0.625 mm) of a healthy male (40 years old, body mass index 22.3). The cortical and cancellous bone of the L4 vertebra, L5 vertebra, and pelvis were segmented from the CT data using Mimics 21.0 (Materialise, Leuven, Belgium) to construct the three-dimensional spine-pelvis model. The extracted lumbar and pelvic structures were imported into Geomagic Studio 17.0(Geomagic, Morrisville, NC, United States) for model reconstruction and smoothing to generate high-quality surface models. Subsequently, joint cartilage and intervertebral discs were created in Solidworks 2021 (Dassault Systemes Corp., Velizy-Villacoublay, France) based on the anatomical structures of the model using Boolean operations (Figure 1).

### 2.2 Construction of the finite element models of denis II type sacral fracture and different fixation methods

**FIGURE 2:**
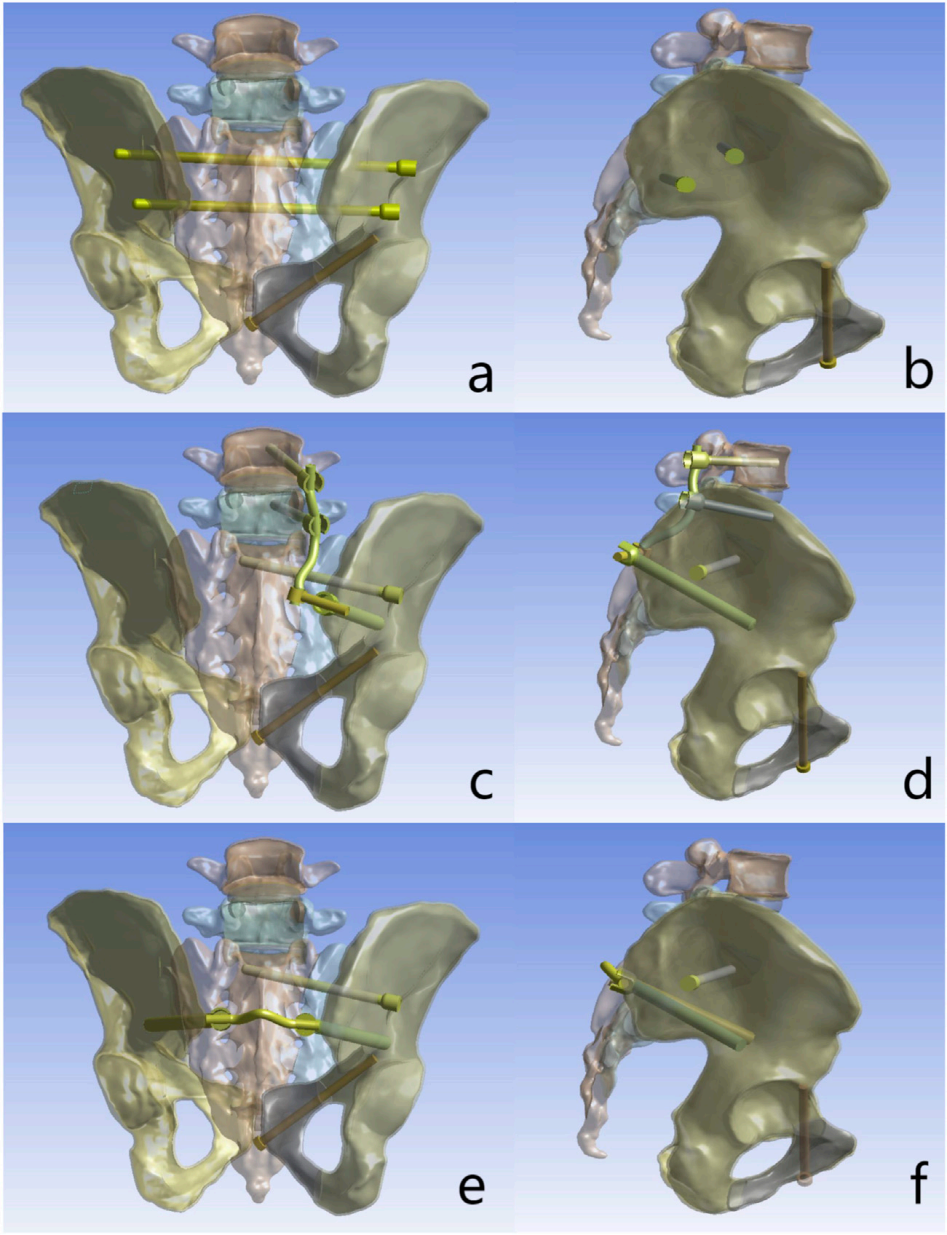
Fixation configurations investigated in this study: **(a)** Posterior view of S1/S2-transiliac-transsacral screws (S1/S2-TTS). **(b)** Lateral view of S1/S2-transiliac-transsacral screws (S1/S2-TTS). **(c)** Posterior view of unilateral L4/5-triangular osteosynthesis (UTOS). **(d)** Lateral view of unilateral L4/5-triangular osteosynthesis (UTOS). **(e)** Posterior view of bilateral S2-alar-iliac (S2AI) screws combined with iliosacral screws (BS2AI-ISS). **(f)** Lateral view of bilateral S2-alar-iliac (S2AI) screws combined with iliosacral screws (BS2AI-ISS).

The intact spino-pelvic model was used to construct a Denis Type II sacral fracture model with intact ligaments (right transforaminal fracture and right superior and inferior pubic ramus fractures) via the split function in Solidworks (Figure 1). In this study, the anterior pelvic ring was fixed with pubic ramus screws in all cases, while three internal fixation techniques were simulated for posterior pelvic ring fixation, including S1/S2-TTS, UTOS, and BS2AI-ISS (Figure 2).

In this study, the diameter of S2AI screws and iliac screws was set to 9 mm. The diameter of lumbar pedicle screws, ISS, TTS, and retrograde pubic ramus screws was 6.5 mm, and the diameter of titanium rods was 6 mm. The length of L4 and L5 pedicle screws was 50 mm; the length of S2AI screws, ISS, iliac screws, and pubic ramus screws was 90 mm; and the length of S1 and S2 TTS was 160 mm and 140 mm, respectively.

### 2.3 Material properties, boundary conditions, and loading

**FIGURE 3:**
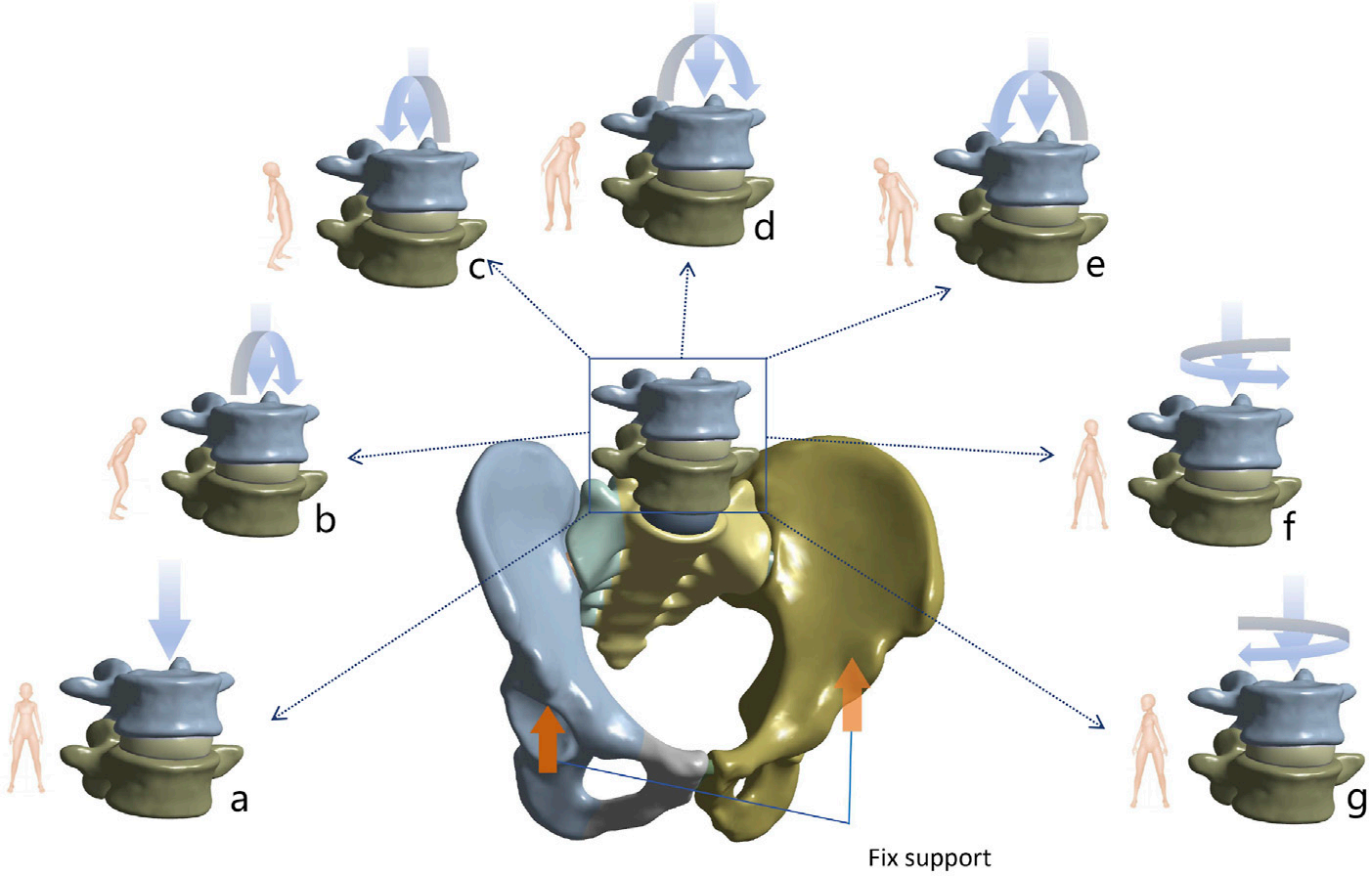
Demonstration of seven different postures for biomechanical analysis. **(a)** Standing **(b)** Flexion **(c)** Extension **(d)** Left flexion **(e)** Right flexion **(f)** Left rotation **(g)** Right rotation.

In Ansys 2021 (ANSYS Inc., Canonsburg, PA, United States), the major ligaments around the lumbopelvic region were simulated using spring ligaments according to previous studies. Cortical bone, cancellous bone, intervertebral discs, articular cartilage, and implants were all meshed with 10-node tetrahedral elements (Ma et al., 2023; Yang et al., 2023; Yang et al., 2025). The cortical bone, cancellous bone, intervertebral discs, joint cartilage, and implants were all meshed using 10-node tetrahedral elements. In the constructed finite element model, the connections between the facet joints, screw head-rod, annulus fibrosus-nucleus pulposus, and cartilaginous endplate-vertebra were modeled using tie constraints. The contact conditions between the sacroiliac joint cartilage, pubic symphysis cartilage, and screws and bone were set as frictional contact with friction coefficients of 0.015, 0.2, and 0.6, respectively. A friction coefficient of 0.3 was applied between the fracture surfaces (Yang et al., 2025). An axial load of 500 N was applied to the upper surface of the L4 vertebra, and the six degrees of freedom of the acetabulum were constrained to simulate the standing position. On this basis, a torque of 7.5 N/m was added to simulate the physiological motions of flexion, extension, left flexion, right flexion, left rotation, and right rotation (Figure 3) (Wang et al., 2021).

### 2.4 Model validation and mesh quality assessment

**FIGURE 4:**
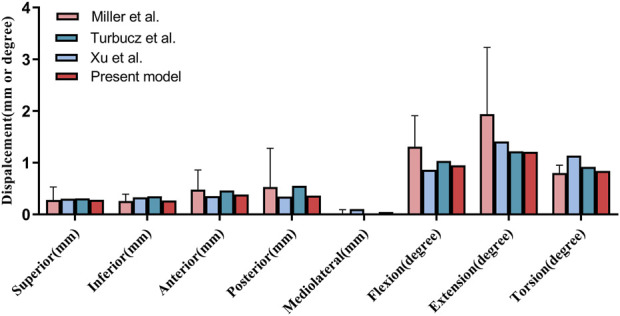
Validation of our finite element model (Present model) via sacral displacement comparison with experimental data by Miller et al., Turbucz et al., and Xu et al. under comparable loads. Error bars indicate one standard deviation.

In this study, the validation of the finite element model was conducted by referring to the methods described by Miller et al. (1987). Specifically, a load of 294 N was applied to the upper surface of the sacral to simulate translational motions in the superior-inferior, anterior-posterior, and medial-lateral directions. Additionally, a moment of 42 N/m was applied to simulate the motion patterns of flexion, extension, and axial rotation. By recording the displacement responses under these loads and moments, the mechanical behavior of the model was evaluated (Figure 4). Ultimately, the finite element simulation results of this study demonstrated high consistency with those of several previously published studies, indicating the model’s good reliability and accuracy (Eichenseer et al., 2011; Xu et al., 2020). The mesh quality of the internal fixation models was assessed, and the results showed that the element quality of all models was above 0.8, indicating that the constructed mesh had high quality and could meet the accuracy requirements for subsequent finite element analysis. Moreover, the number of elements and nodes for each model is presented in Table 1.

**TABLE 1:** Number of elements and nodes and elements quality in different models.

Model	Intact	S1/S2-TTS	UTOS	BS2AI-ISS
Nodes	1931776	1959389	1966024	1956623
Elements	1241086	1251111	1254400	1248793
Elements quality	0.8307	0.8289	0.8294	0.8295

### 2.5 Finite element analysis

**FIGURE 5:**
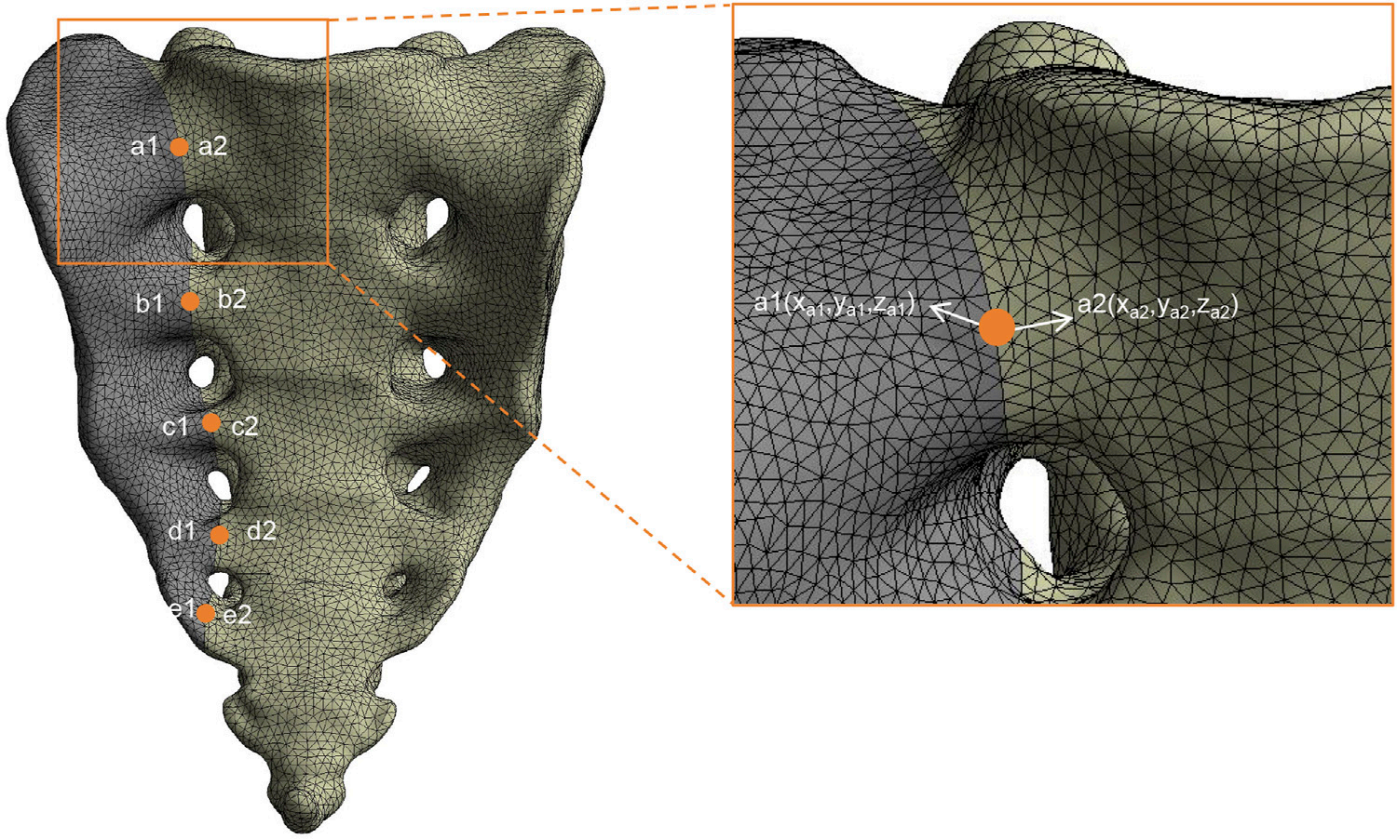
Finite element model with five pairs of observation points for the evaluation of relative interfragmentary displacement (RID). An enlarged view highlights a pair of observation points, a1 (x_a1_, y_a1_, z_a1_) and a2 (x_a2_, y_a2_, z_a2_), utilized for measuring RID.

This study evaluated different sacral fracture fixation techniques under seven motion states: standing, flexion, extension, left flexion, right flexion, left rotation, and right rotation. The vertical stiffness (VS) of the sacrum in the standing position was calculated by dividing the load by the vertical displacement of the center point on the upper surface of the sacral, using the formula: VS = Load (N)/Displacement (mm). The VS of each fixation model was normalized to that of the intact spine-pelvis and presented as a percentage, with the VS of the intact spine-pelvis set at 100%. Five pairs of observation points were set along the fracture line on the anterior surface of the sacral, and the relative interfragmentary displacement (RID) was assessed using the three-dimensional coordinates (x, y, z) of each pair of points, with the calculation formula: RID = $\sqrt{{\left(x_{1}-x_{2}\right)}^{2}+{\left(y_{1}-y_{2}\right)}^{2}+{\left(z_{1}-z_{2}\right)}^{2}}$ (Figure 5). Other evaluation variables include the maximum Von Mises stress of bone and implants, as well as the stress shielding phenomenon.

### 2.6 Statistical analysis

Data collection and management were conducted using Microsoft Excel 2019. For statistical analysis, SPSS version 25.0 (SPSS, Chicago, IL, United States) was employed. Descriptive statistics were computed to summarize the variables, which were expressed as mean ± standard deviation (SD). To determine if there were significant differences among groups, we performed one-way Analysis of Variance (ANOVA). In cases where ANOVA indicated significant differences (p < 0.05), we conducted *post hoc* analysis using the Least Significant Difference (LSD) test to identify which specific group means differed from each other. The level of statistical significance was set at p < 0.05 for all tests.

## 3 Results

### 3.1 The VS of each model

**FIGURE 6:**
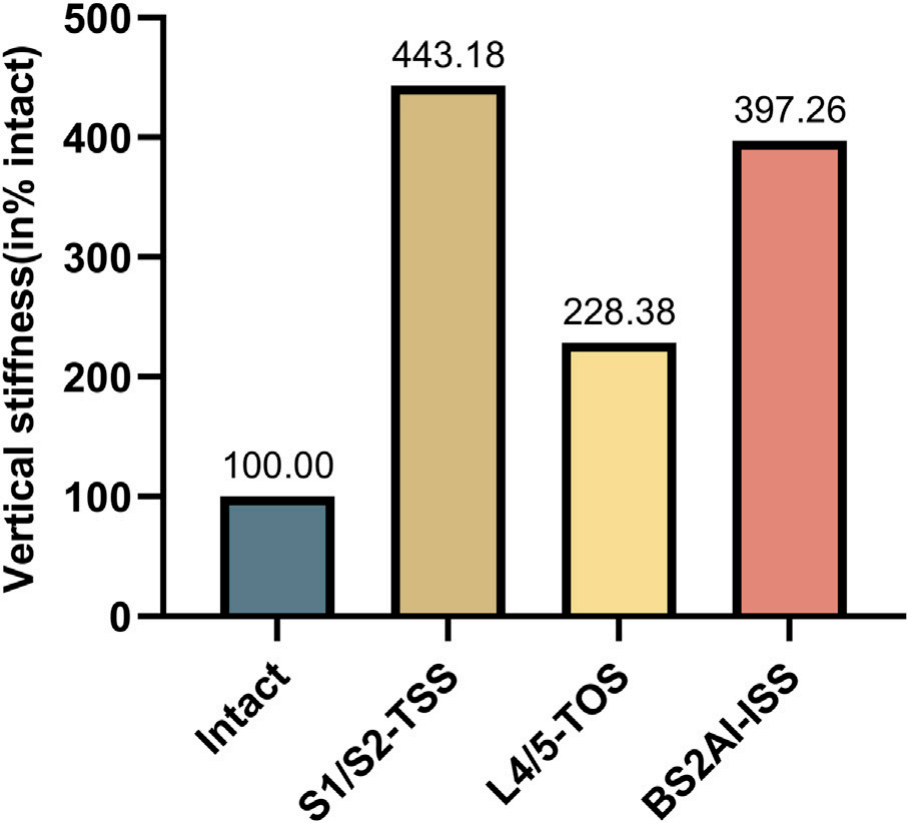
Normalized vertical stiffness of different internal fixation techniques.

In this study, the VS of each fixation model was normalized to the VS of the intact spino-pelvic structure, presented as a percentage with the VS of the intact spino-pelvic structure set as 100%. The results showed that all fixation techniques significantly increased the VS of the sacrum. Specifically, the VS of the sacrum in the S1/S2-TTS, UTOS, and BS2AI-ISS models increased to 443.18%, 228.38%, and 397.26%, respectively (Figure 6).

### 3.2 Maximum Von Mises stress of the implants

**FIGURE 7:**
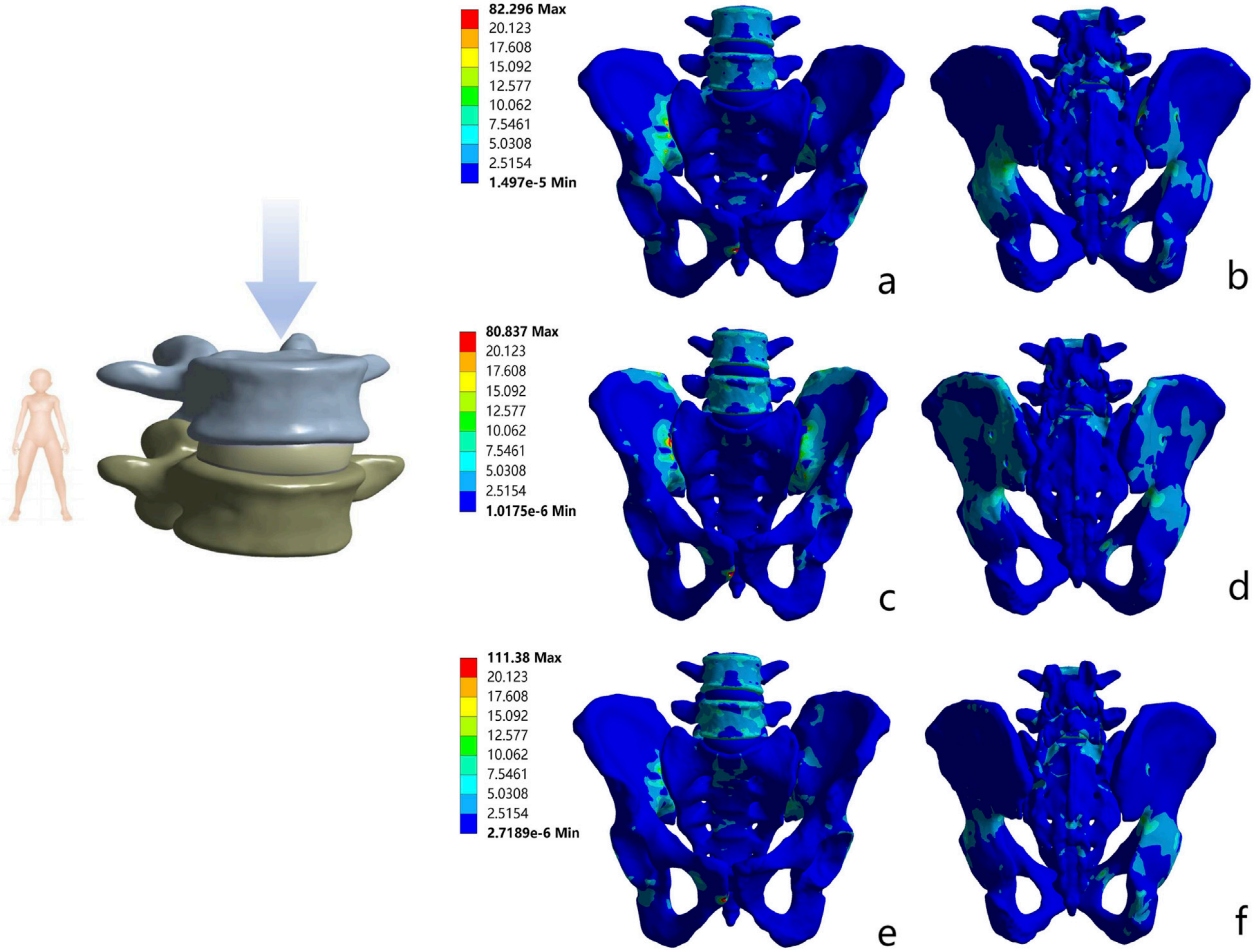
Von Mises stress distribution of pelvic bones with three internal fixations in standing position. **(a)** Anterior view of S1/S2 - TTS; **(b)** Posterior view of S1/S2 - TTS; **(c)** Anterior view of UTOS; **(d)** Posterior view of UTOS; **(e)** Anterior view of BS2AI - ISS; **(f)** Posterior view of BS2AI - ISS.

In all motion states, the maximum Von Mises stress levels of the internal fixators in all models were lower than the yield strength of titanium alloy. Further analysis revealed that the S1/S2-TTS model exhibited the lowest stress values in all test states, with the maximum Von Mises stress ranging from 30.30 MPa to 49.23 MPa. The stress values in the BS2AI-ISS model were intermediate, ranging from 51.67 MPa to 76.47 MPa. In contrast, the UTOS model showed the highest stress values, with the maximum Von Mises stress ranging from 110.41 MPa to 329.01 MPa (Figure 7; Supplementary Table S1).

### 3.3 Stress shielding phenomenon

In all motion states, the stress distribution of the three models was transmitted along the iliopubic line. For the S1/S2-TTS, UTOS, and BS2AI-ISS models, the maximum Von Mises stress ranges of the bone tissue were 80.76–80.87 MPa, 82.25–82.35 MPa, and 111.34–111.42 MPa, respectively, with all maximum stress values being lower than the yield strength of bone.

**FIGURE 8:**
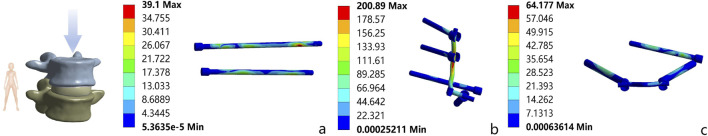
Von Mises stress distribution of three internal fixation constructs in standing position. **(a)** S1/S2 - TTS; **(b)** UTOS; **(c)** BS2AI - ISS.

Meanwhile, to evaluate the biocompatibility of the implants, this study analyzed the stress shielding effect of the implants on sacral fractures. The maximum stress difference between the implant and the injured sacrum can be used to characterize the degree of the stress shielding phenomenon. In the standing position, the maximum stress differences between the implant and the injured sacrum in the S1/S2-TTS, UTOS, and BS2AI-ISS models were 14.74 MPa, 183.43 MPa, and 19.59 MPa, respectively. In other motion states, the maximum stress differences between the implant and the injured sacrum showed a consistent trend with that in the standing state (Figure 8; Supplementary Table S2).

### 3.4 RID at the observation points on the anterior surface of the sacral fracture line

In the standing position, the mean RID in the S1/S2-TTS, UTOS, and BS2AI-ISS models were 0.0313 ± 0.0148 mm, 0.0736 ± 0.0314 mm, and 0.0539 ± 0.0163 mm, respectively. The LSD *post hoc* test showed that only the difference between S1/S2-TTS and UTOS was statistically significant (p = 0.047), while the differences between BS2AI-ISS and S1/S2-TTS, and between BS2AI-ISS and UTOS did not reach a significant level (p = 0.352 and p = 0.441, respectively).

**FIGURE 9:**
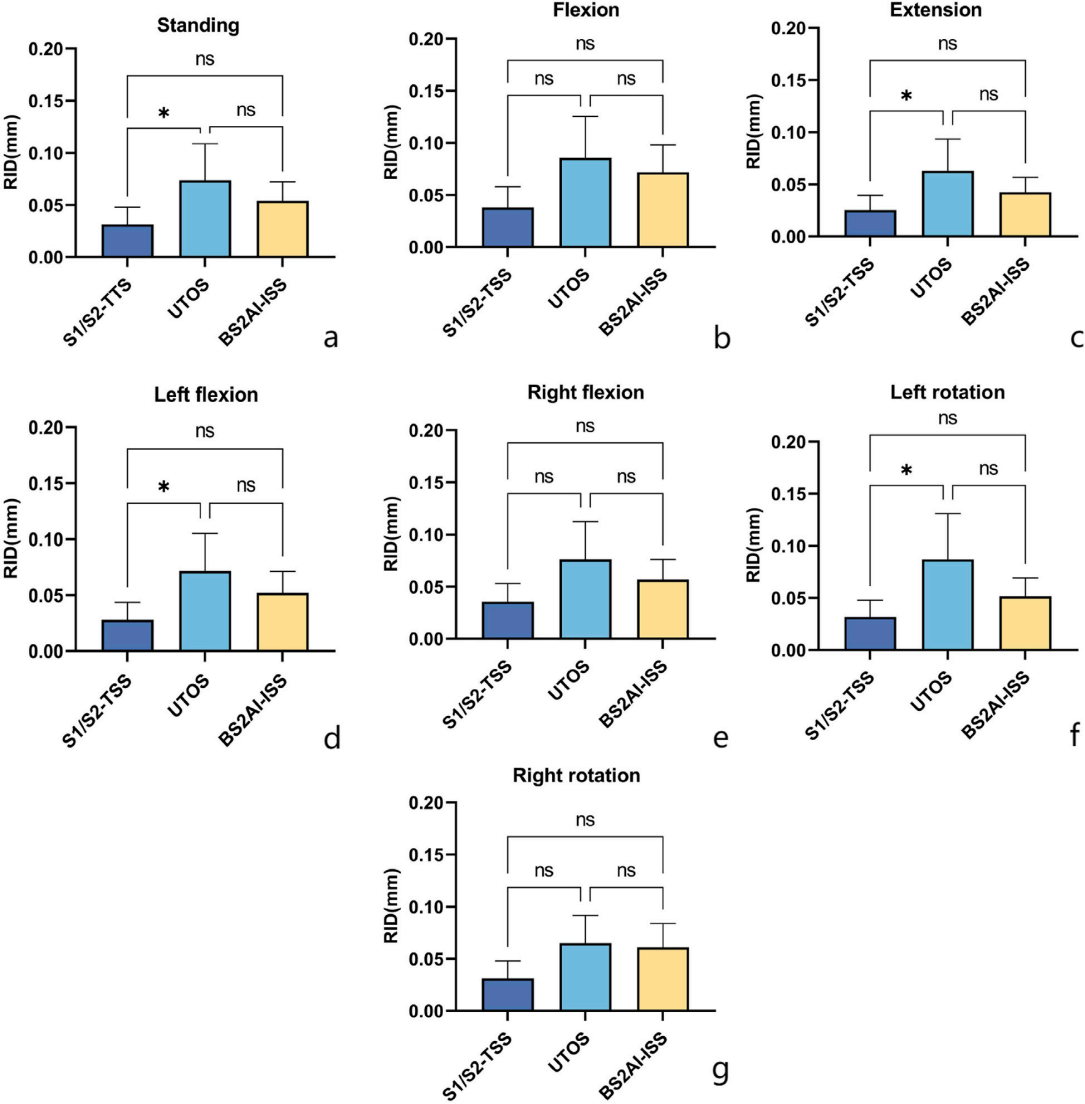
Posture - specific RID (Relative interfragmentary displacement) comparison of three internal fixations: Insights from LSD post - hoc analysis. Seven postural conditions: **(a)** Standing; **(b)** Flexion; **(c)** Extension; **(d)** Left flexion; **(e)** Right flexion; **(f)** Left rotation; **(g)** Right rotation. One - way ANOVA with LSD pairwise comparisons was performed; “*” denotes p < 0.05 (significant), “ns” denotes non - significant results.

In the conditions of extension, left flexion, and left rotation, the above difference pattern was consistent with that in the standing position; in the conditions of right flexion and right rotation, there were no statistically significant differences in RID among the three models (Figure 9).

## 4 Discussion

Sacral fractures are a rare and challenging type of fracture, accounting for 28%–45% of all pelvic fractures, among which unstable fractures account for 17%–30% (Denis et al., 1988; Taguchi et al., 1999). Surgical intervention is the preferred treatment method, as it can both restore and stabilize the normal anatomical structure of the sacrum and prevent sequelae of malunion of the sacrum (Santolini et al., 2020). The key to sacral fracture repair is that the implant can achieve good stability in both vertical and horizontal directions while minimizing adverse effects on the patient, thereby promoting early mobilization and rehabilitation (Zheng et al., 2023).

The VS of the sacrum is one of the key indicators used to evaluate its ability to resist deformation under vertical loads (Acklin et al., 2018). Turbucz et al. (2023) assessed the fixation of sacral Denis Type II fractures with different combinations of ISS and TTS and found that the use of TTS provided higher stability, while 2 TTS screws were superior to all other internal fixation combinations, with the greatest vertical stability, minimal inter-fragment displacement and implant stress. The results of this study showed that all three different internal fixation models significantly increased the VS of the sacrum. The VS of the BS2AI-ISS model was slightly lower than that of the S1/S2-TTS model, but the VS of both models was much higher than that of the UTOS fixation model. This result indicates that the BS2AI-ISS model has similar stability to the S1/S2-TTS model in controlling the deformation of the injured sacral fracture.

In biomechanical studies, the maximum Von Mises stress of bone and implants is an important indicator for evaluating the risk of secondary fractures and implant fractures (Frost, 1997). Böhme et al. (2012), through finite element (FE) model analysis combined with radiological examinations during clinical rehabilitation, found that the locations of implant loosening and secondary fractures were highly consistent with the areas with the highest stress levels. The results of this study showed that the maximum von Mises stress of the bony structures corresponding to the three internal fixation models was significantly lower than the yield threshold of cancellous bone. The stress nephogram further revealed that under different loading conditions, the stress trajectory continued along the iliopubic arch and spread uniformly, with no abnormal stress concentration. It can be inferred that all three fixation methods can effectively reconstruct the physiological load transmission channel of the pelvis, significantly reduce the risk of postoperative secondary bone fractures, and their mechanical behavior conforms to the biomechanical principles of pelvic stability reconstruction (Liu et al., 2016a).

In addition, in all motion states including the standing position, the maximum stress values of the implants in the three models of internal fixation models did not exceed the yield stress of titanium alloy, indicating that the risk of implant fracture and failure can be ignored under static and regular motion states (Ma et al., 2023). However, the study also found that the implant stress in the UTOS model was much higher than that in the other two models in all postures. This result indicates that the risk of implant fatigue damage and screw loosening in the S1/S2-TTS model and BS2AI-ISS model is lower than that in the UTOS model. We speculate that the S1/S2-TTS model and BS2AI-ISS model not only fixed the bilateral sacrum but also the bilateral ilium, having a suspension bridge structure similar to the sacroiliac complex, thereby making the stress of the implant more dispersed.

Based on the stress shielding theory, the smaller the stress difference between the pelvis and the implant, the better the biocompatibility of the implant. Hu et al. (2019) constructed a finite element model for the treatment of sacral fractures using a tension band plate or 2 sacroiliac screws, and the results showed that the biomechanical compatibility of the model fixed with 2 sacroiliac screws was significantly better than that of the model with the tension band plate. In this study, under different motion states, the biomechanical compatibility of the BS2AI-ISS model was slightly inferior to that of the S1/S2-TTS model, but much better than that of the UTOS model. It is speculated that the reason for this phenomenon may be that the UTOS model adopts a trans-lumbosacral fixation method. During movement, the implant is prone to stress concentration in the lumbosacral region, resulting in a higher maximum Von Mises stress, which in turn makes the stress shielding phenomenon in the UTOS model more significant than in the other two models.

Based on the biological principles of fracture healing, intermittent movement (micromotion) between fracture ends can promote callus formation and accelerate fracture healing (Claes, 1989; Woo et al., 1984). Studies have shown that such micromotion should not exceed 1 mm, as exceeding this limit will have a negative impact on fracture healing (Claes, 2011; Goodship and Kenwright, 1985). In this study, the fracture displacement values of the three internal fixation models under different motion modes did not exceed 1 mm, indicating that all three internal fixation models can provide a relatively stable healing environment for the injured sacrum. Zheng et al. (Zheng et al., 2021) found that under a vertical load of 500 N, the RID displacement of sacral Denis Type II fractures fixed with 2 TTS screws was lower than that of the UTOS model, which is consistent with the results of this study. Further inter-model comparison revealed that the RID of the UTOS model was statistically significant compared with that of the S1/S2-TTS model in standing, hyperextension, left lateral flexion, and left rotation, which indicates that the S1/S2-TTS model is superior to the UTOS model in maintaining the stability of sacral fractures. For the BS2AI-ISS model, the study found that although its RID was lower than that of the S1/S2-TTS model in all postures, there was no significant statistical difference between the two models. This indicates that this structure can effectively transfer the load from the lumbar spine to the bilateral ilium through the internal fixation system in any posture, thereby maintaining the stability of the sacral fracture.

In summary, the proposed internal fixation combination of bilateral S2AI screws combined with unilateral sacroiliac screws has the following potential advantages: 1. Wide anatomical indications: For patients with TTS/ISS transosseous corridor obstruction (e.g., Tarlov cysts, tumors), our technique offers a viable alternative—one potentially more effective than in sacral dysmorphism—by bypassing pathological bony routes. 2. Preservation of lumbosacral mobility: The fixation range ends at the S1 vertebra, without the need for transarticular fixation of the lumbar spine, which theoretically can reduce the risk of lumbosacral stiffness and adjacent segment degeneration. 3. Excellent mechanical properties: Finite element analysis shows that compared with the classic S1/S2-TTS and UTOS, the fixation efficacy of BS2AI-ISS for unilateral Denis Type II sacral fractures has no statistically significant differences in key biomechanical indicators such as maximum internal fixation stress and relative displacement of fracture, and the three have equal immediate stability.

This study still has some limitations. Firstly, the titanium rod between the bilateral S2AI screws may cause skin irritation, leading to complications such as skin pain and necrosis. The connection between the titanium rod and the screw head cannot improve the acute angle between the screw head and the screw, and previous studies have shown that the acute angle between the screw head and the screw is a potential failure point. Secondly, the bone homogenization model used in this study does not consider bone mineral density, and the osteoporotic model may cause greater displacement. Furthermore, although studies have confirmed that both pubic ramus screws and plates can stabilize the anterior pelvic ring, this study mainly focuses on sacral fractures and does not analyze pubic ramus fractures. Finally, we mainly used static loads, and although various postures were simulated, the impact of long-term biomechanics could not be discussed in depth. Future cadaveric biomechanical experiments and clinical studies are still needed for further verification.

## 5 Conclusion

The present study evaluated the biomechanical stability of three internal fixation methods, namely, S1/S2-TTS, UTOS, and BS2AI-S1, in unilateral sacral Denis Type II fractures. The results showed that all three methods could achieve good biomechanical stability, with S1/S2-TTS being the highest, BS2AI-ISS being moderate, and UTOS being the lowest. BS2AI-ISS can still obtain good biomechanical stability without affecting lumbar spine movement and being restricted by sacral morphology, suggesting that BS2AI-ISS may be an alternative option for the treatment of unilateral sacral Denis Type II fractures.

## Data Availability

The raw data supporting the conclusions of this article will be made available by the authors, without undue reservation.

## Abbreviations

S2AI, S2-alar-iliac; ISS, Iliosacral screw; S1/S2-TTS, S1/S2-transiliac-transsacral screws; TOS, Triangular osteosynthesis; UTOS, Unilateral L4/5-triangular osteosynthesis; BS2AI-ISS, Bilateral S2AI screws combined with iliosacral screws; VS, Vertical Stiffness; RID, Relative interfragmentary displacement; FE, Finite element.
