# Supplementary material for: A biomechanical investigation of three fixation methods for unilateral denis type II sacral fractures using finite element analysis
Source: Front Bioeng Biotechnol. 2025 Aug 25;13:1631457. doi: 10.3389/fbioe.2025.1631457 (PMC12415403; doi:10.3389/fbioe.2025.1631457)
Supplement: Supplementary file 1 [file Table1.docx]

|  | Intact | S1/S2-TTS | UTOS | BS2AI-ISS |
| --- | --- | --- | --- | --- |
| Nodes | 1852475 | 1872034 | 1884211 | 1880338 |
| Elements | 1180166 | 1186169 | 1191913 | 1190550 |
| Elements quality | 0.8326 | 0.8212 | 0.8311 | 0.8308 |

**Table 1.** Number of elements and nodes and elements quality in different models.
