# Supplementary material for: A biomechanical investigation of three fixation methods for unilateral denis type II sacral fractures using finite element analysis
Source: Front Bioeng Biotechnol. 2025 Aug 25;13:1631457. doi: 10.3389/fbioe.2025.1631457 (PMC12415403; doi:10.3389/fbioe.2025.1631457)
Supplement: Supplementary file 2 [file DataSheet2.docx]

| Suppl 2 Fig 1. Von Mises stress distribution of three internal fixation constructs in flexion position. (a) S1/S2 - TTS; (b) UTOS 2; (c) BS2AI - ISS. |
| --- |
| 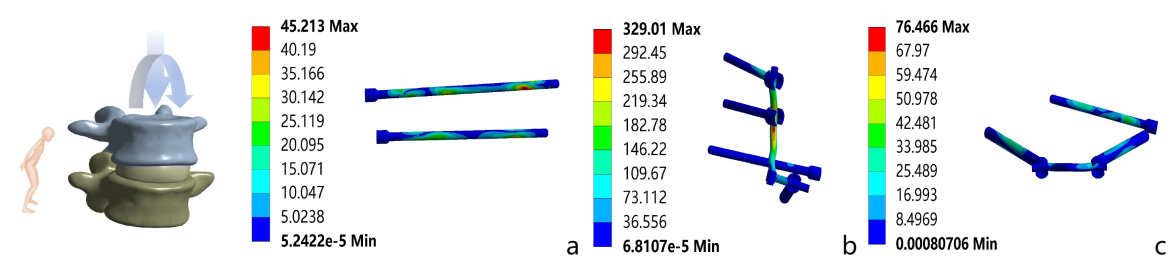 |
| Suppl 2 Fig 2. Von Mises stress distribution of three internal fixation constructs in extension position. (a) S1/S2 - TTS; (b) UTOS 2; (c) BS2AI - ISS. |
| 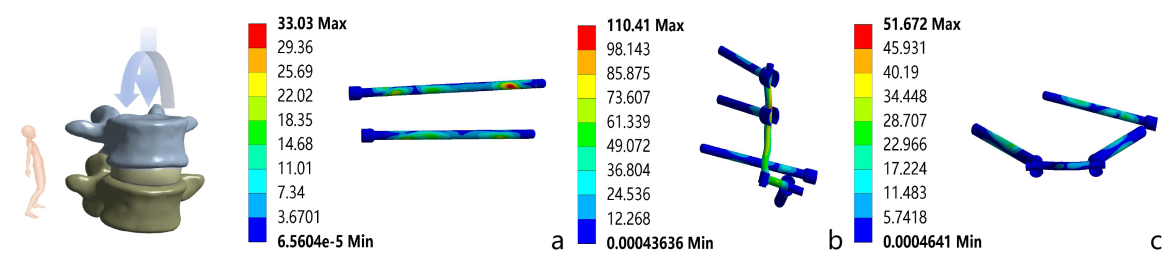 |
| Suppl 2 Fig 3. Von Mises stress distribution of three internal fixation constructs in left flexion position. (a) S1/S2 - TTS; (b) UTOS 2; (c) BS2AI - ISS. |
| 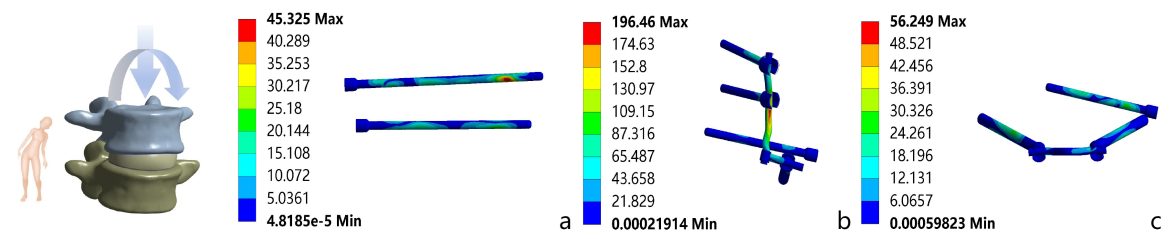 |
| Suppl 2 Fig 4. Von Mises stress distribution of three internal fixation constructs in right flexion position. (a) S1/S2 - TTS; (b) UTOS 2; (c) BS2AI - ISS. |
| 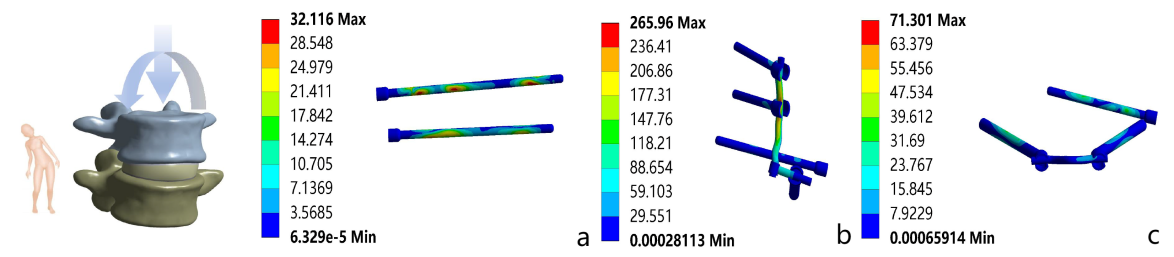 |
| Suppl 2 Fig 5. Von Mises stress distribution of three internal fixation constructs in left rotation position. (a) S1/S2 - TTS; (b) UTOS 2; (c) BS2AI - ISS. |
| 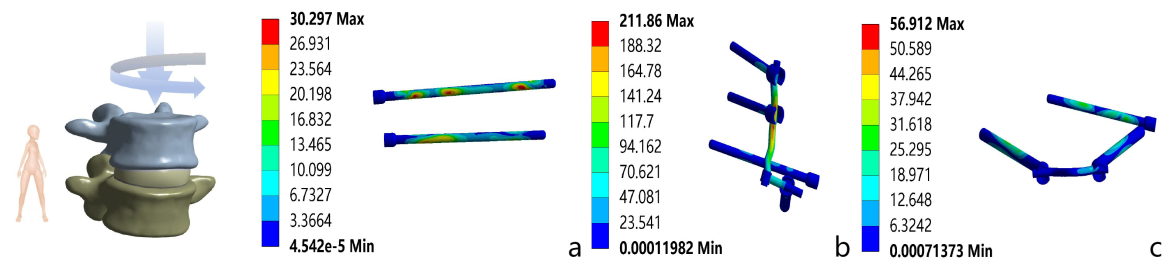 |
| Suppl 2 Fig 6. Von Mises stress distribution of three internal fixation constructs in right rotation position. (a) S1/S2 - TTS; (b) UTOS 2; (c) BS2AI - ISS. |
| 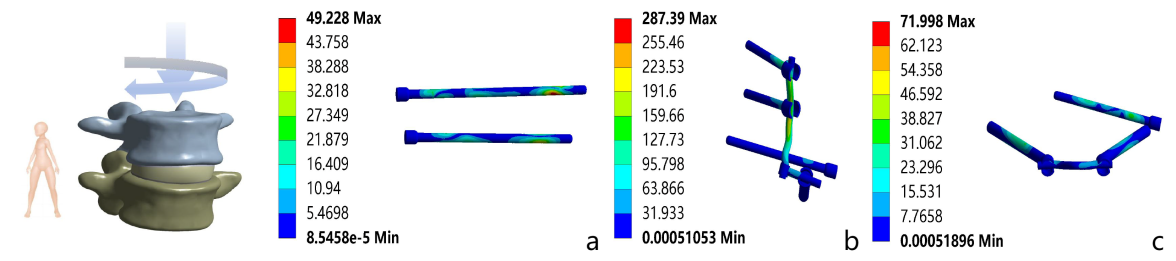 |
|  |
