# Supplementary material for: A biomechanical investigation of three fixation methods for unilateral denis type II sacral fractures using finite element analysis
Source: Front Bioeng Biotechnol. 2025 Aug 25;13:1631457. doi: 10.3389/fbioe.2025.1631457 (PMC12415403; doi:10.3389/fbioe.2025.1631457)
Supplement: Supplementary file 3 [file DataSheet1.docx]

| Suppl 1 Fig 1. Von Mises stress distribution of pelvic bones with three internal fixations in flexion position. (a) Anterior view of S1/S2 - TTS; (b) Posterior view of S1/S2 - TTS; (c) Anterior view of UTOS; (d) Posterior view of UTOS; (e) Anterior view of BS2AI - ISS; (f) Posterior view of BS2AI - ISS. |
| --- |
| 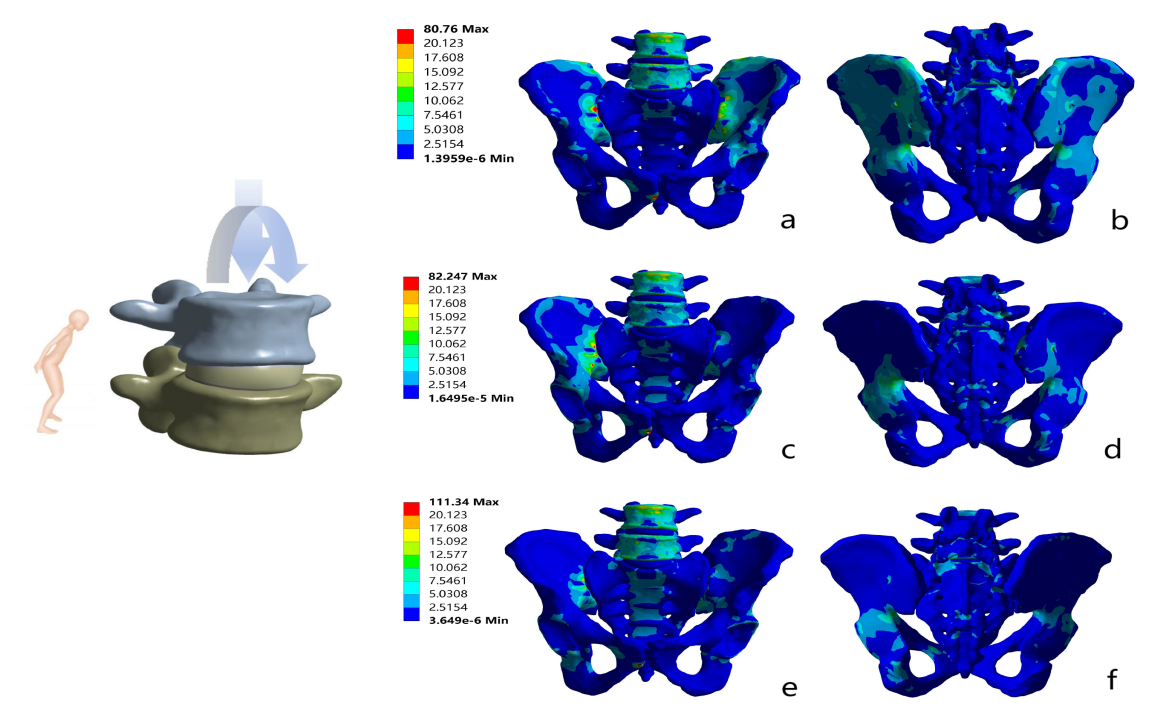 |
| Suppl 1 Fig 2. Von Mises stress distribution of pelvic bones with three internal fixations in extension position. (a) Anterior view of S1/S2 - TTS; (b) Posterior view of S1/S2 - TTS; (c) Anterior view of UTOS; (d) Posterior view of UTOS; (e) Anterior view of BS2AI - ISS; (f) Posterior view of BS2AI - ISS. |
| 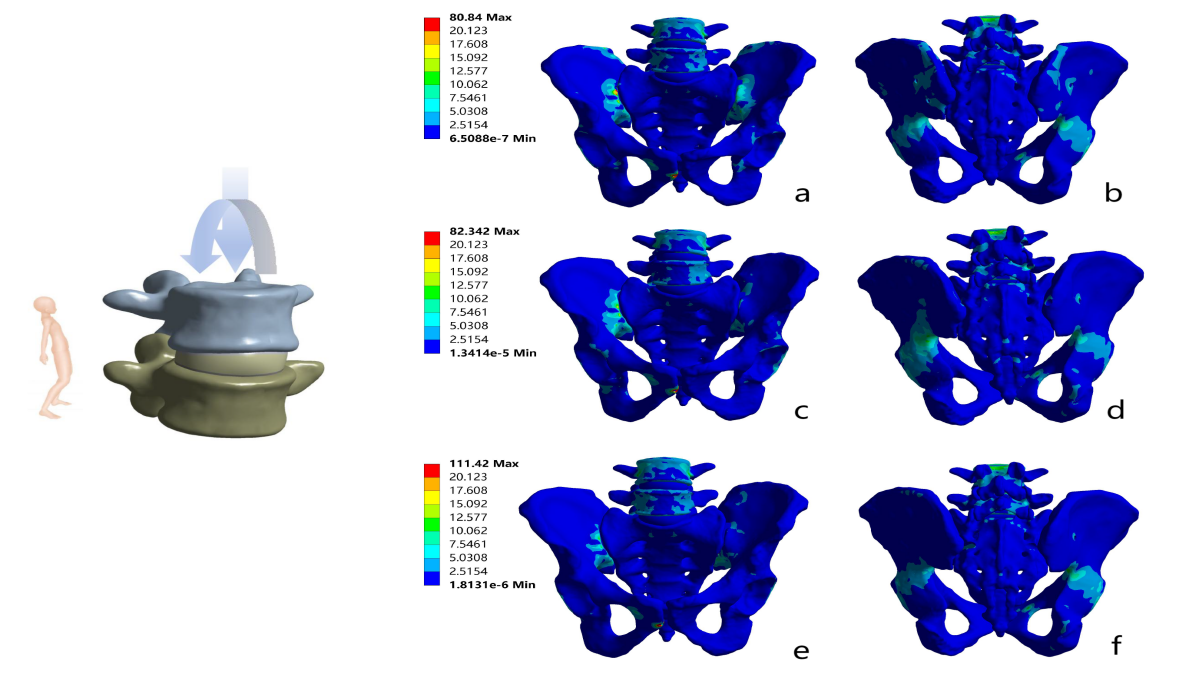 |

| Suppl 1 Fig 3. Von Mises stress distribution of pelvic bones with three internal fixations in Left flexion position. (a) Anterior view of S1/S2 - TTS; (b) Posterior view of S1/S2 - TTS; (c) Anterior view of UTOS; (d) Posterior view of UTOS; (e) Anterior view of BS2AI - ISS; (f) Posterior view of BS2AI - ISS. |
| --- |
| 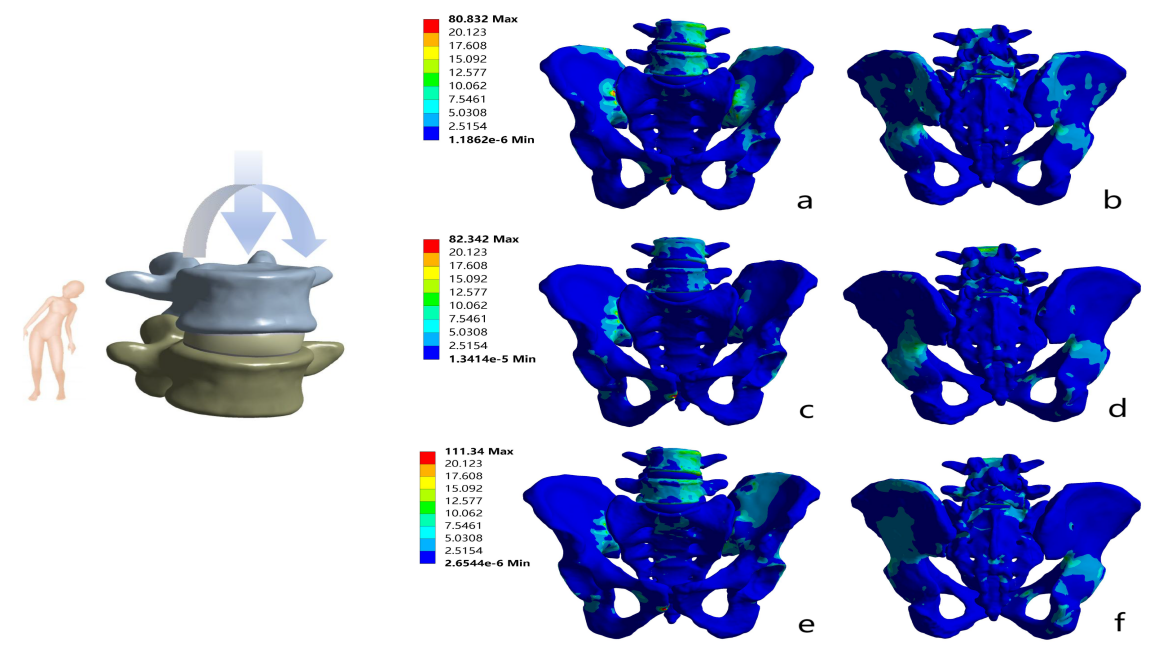 |
| Suppl 1 Fig 4. Von Mises stress distribution of pelvic bones with three internal fixations in right flexion. (a) Anterior view of S1/S2 - TTS; (b) Posterior view of S1/S2 - TTS; (c) Anterior view of UTOS; (d) Posterior view of UTOS; (e) Anterior view of BS2AI - ISS; (f) Posterior view of BS2AI - ISS. |
| 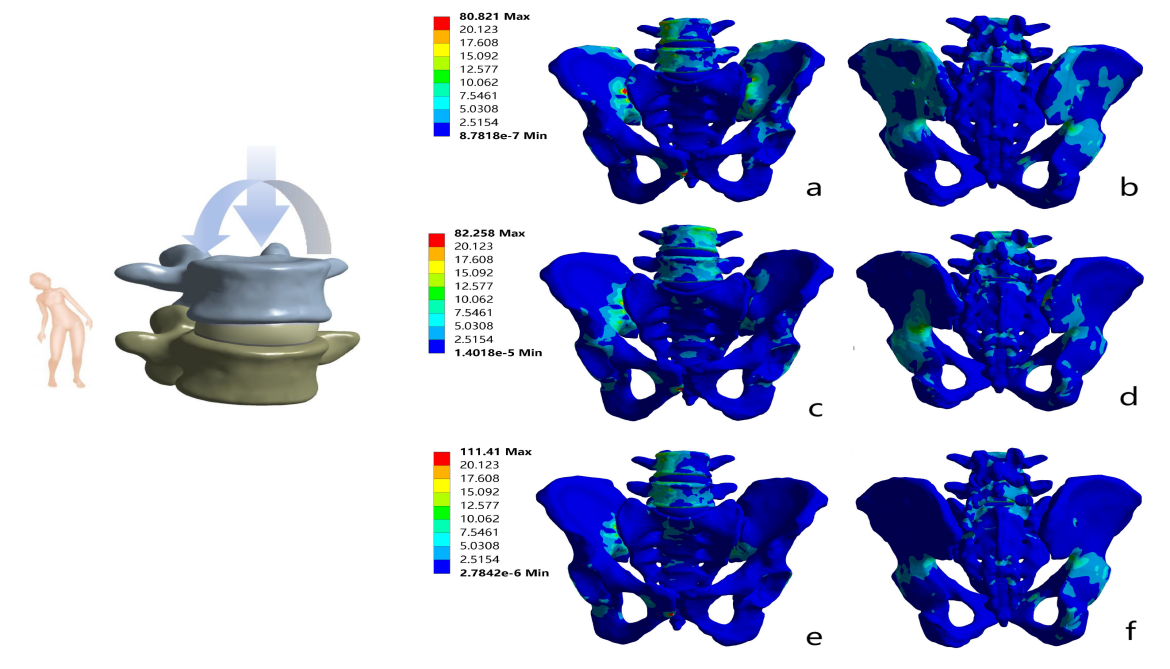 |

| Suppl 1 Fig 5. Von Mises stress distribution of pelvic bones with three internal fixations in left rotation position. (a) Anterior view of S1/S2 - TTS; (b) Posterior view of S1/S2 - TTS; (c) Anterior view of UTOS; (d) Posterior view of UTOS; (e) Anterior view of BS2AI - ISS; (f) Posterior view of BS2AI - ISS. |
| --- |
| 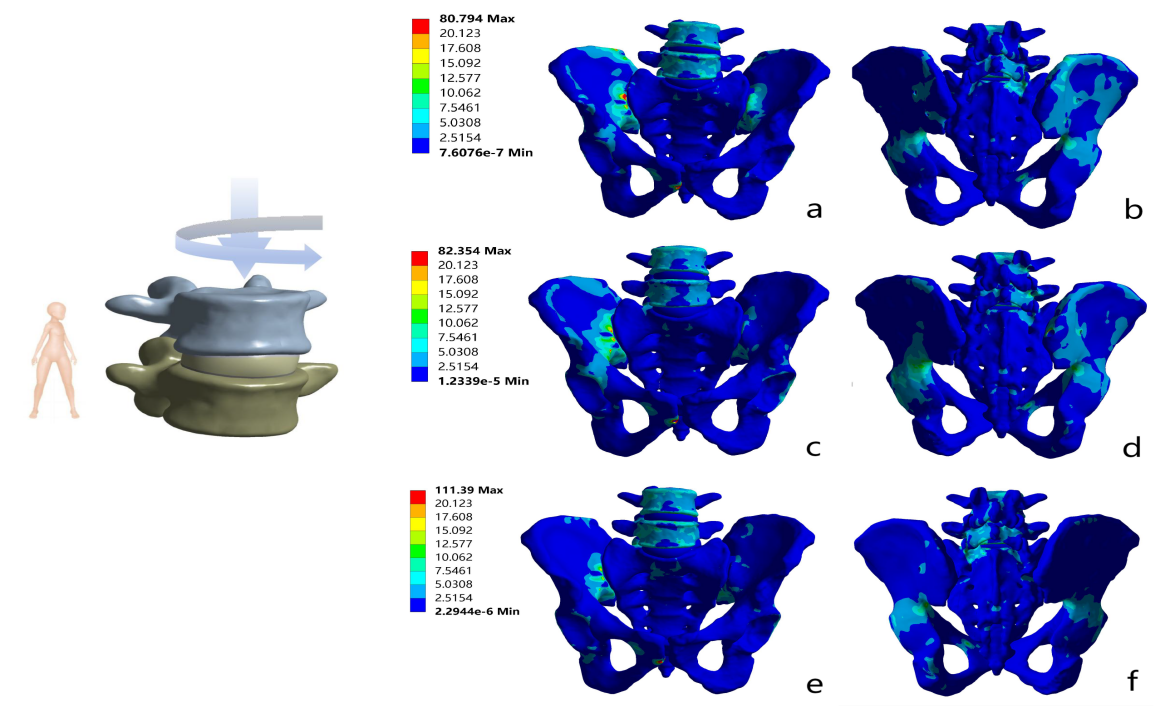 |
| Suppl 1 Fig 6. Von Mises stress distribution of pelvic bones with three internal fixations in right rotation position. (a) Anterior view of S1/S2 - TTS; (b) Posterior view of S1/S2 - TTS; (c) Anterior view of UTOS; (d) Posterior view of UTOS; (e) Anterior view of BS2AI - ISS; (f) Posterior view of BS2AI - ISS. |
| 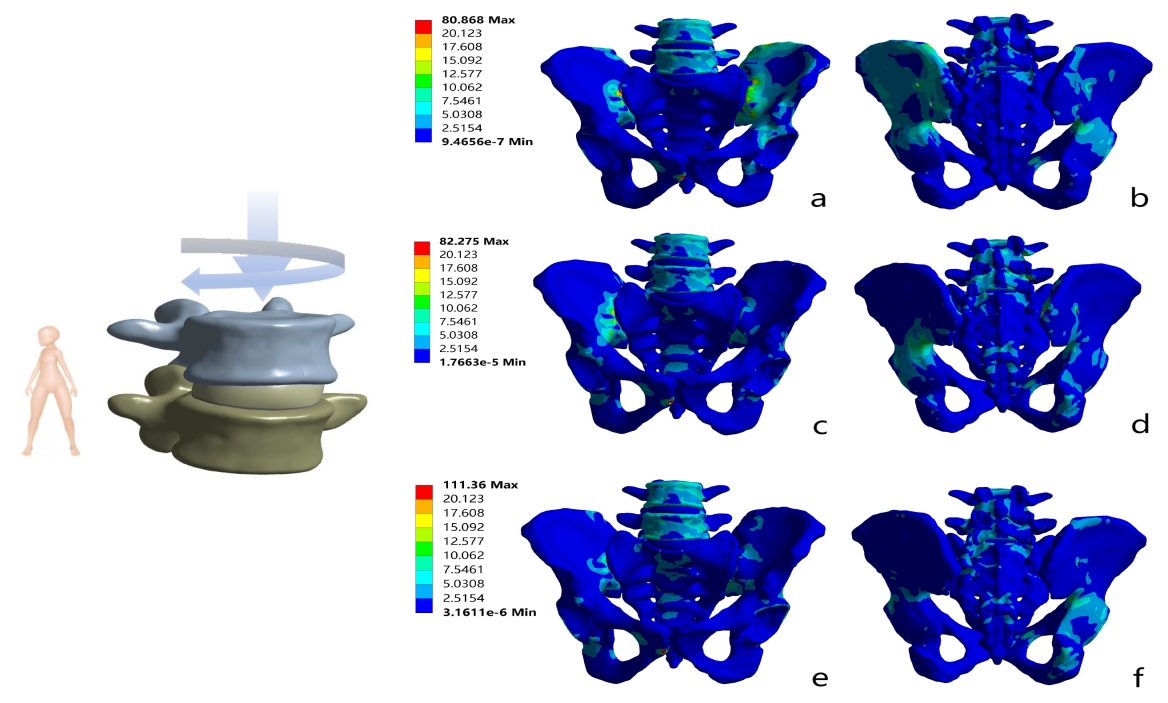 |
